# An integrated SII-PNI immune-nutritional scoring system predicts efficacy and immune-related adverse events in locally advanced gastric cancer patients undergoing neoadjuvant immunotherapy

**DOI:** 10.3389/fimmu.2026.1806537

**Published:** 2026-04-30

**Authors:** Fang Li, Honghai Guo, Haotian Wu, Jiaxiang Wu, Shuo Ma, Yihao Yang, Yuan Tian, Wenqian Ma, Qun Zhao

**Affiliations:** 1 Department of Pathology, The Fourth Hospital of Hebei Medical University, Shijiazhuang, Hebei, China; 2 The Third Department of Surgery, the Fourth Hospital of Hebei Medical University, Shijiazhuang, Hebei, China; 3 Hebei Key Laboratory of Precision Diagnosis and Comprehensive Treatment of Gastric Cancer, Shijiazhuang, Hebei, China; 4 Big Data Analysis and Mining Application for Precise Diagnosis and Treatment of Gastric Cancer Hebei Provincial Engineering Research Center, Shijiazhuang, Hebei, China; 5 Department of Endoscopy, The Fourth Hospital of Hebei Medical University, Shijiazhuang, China

**Keywords:** gastric cancer, immune-related adverse events, neoadjuvant immunotherapy, prognostic nutritional index, systemic immune-inflammation index

## Abstract

**Background:**

Immune checkpoint inhibitors (ICIs) have transformed treatment of locally advanced gastric cancer (LAGC), yet response heterogeneity and immune-related adverse events (irAEs) remain major challenges. Systemic inflammation and nutritional status critically modulate antitumor immunity. This study evaluated the dual predictive value of a combined Systemic Immune-Inflammation Index and Prognostic Nutritional Index (SII-PNI) score for therapeutic efficacy and safety in LAGC patients receiving neoadjuvant PD-1/PD-L1 inhibitor-based therapy.

**Methods:**

We retrospectively analyzed 276 LAGC patients who received PD-1/PD-L1 inhibitor-based neoadjuvant immunotherapy (January 2019–December 2021). Blood samples were collected within 7 days before treatment initiation. Optimal cut-off values for SII and PNI were determined via ROC analysis to construct the SII-PNI score (Score 0–2). Primary endpoints were disease-free survival (DFS), overall survival (OS), tumor response (RECIST 1.1), and irAE incidence.

**Results:**

The optimal cut-off values were 736.2 for SII and 48.5 for PNI. Patients with a high SII-PNI score (Score 2) exhibited significantly poorer objective response rates (ORR) compared to the low-risk Score 0 group (22.6% vs. 71.2%, P < 0.001). The SII-PNI score demonstrated superior predictive accuracy for immunotherapy response (AUC = 0.750) compared to PD-L1 CPS (AUC = 0.711) and Tumor Mutational Burden (TMB, AUC = 0.625). Multivariate Cox analysis identified the SII-PNI score as an independent prognostic factor; compared to Score 0, patients with Score 2 faced a more than threefold increased risk of disease recurrence (HR = 3.45, 95% CI: 2.40–4.95, P < 0.001) and mortality (HR = 3.15, 95% CI: 1.85–5.36, P < 0.001). Furthermore, the high-risk group (Score 2) had a significantly higher incidence of severe (Grade 3–4) irAEs compared to the low-risk group (28.6% vs. 3.6%, P < 0.001).

**Conclusions:**

The SII-PNI score is a robust, non-invasive, and economically accessible biomarker that effectively stratifies outcomes in gastric cancer patients receiving neoadjuvant immunotherapy. It serves as a dual predictor for both poor therapeutic response and increased toxicity, outperforming traditional biomarkers like PD-L1 CPS. This scoring system holds promise for guiding personalized treatment strategies and optimizing patient selection.

## Introduction

Gastric cancer remains a leading cause of cancer-related mortality worldwide, with a particularly high burden in East Asia (1, 2). While traditional chemotherapy has long been the cornerstone of treatment for advanced disease, its efficacy has reached a plateau (3–5). The advent of immune checkpoint inhibitors (ICIs), particularly those targeting programmed cell death protein 1 (PD-1) and its ligand (PD-L1), has revolutionized the therapeutic landscape (6, 7). Landmark Phase III clinical trials, most notably CheckMate 649 (8) and ATTRACTION-4 (9), have demonstrated that PD-1 inhibitors combined with chemotherapy can significantly prolong overall survival and progression-free survival compared to chemotherapy alone, thereby establishing a new standard of care for advanced gastric cancer. However, the clinical reality remains challenging: only a fraction of patients derive durable benefits from ICIs, while others experience rapid disease progression or severe immune-related adverse events (irAEs). Therefore, identifying robust biomarkers to precisely select patients who are most likely to benefit from immunotherapy is a critical unmet need in current oncological practice.

Currently, established biomarkers such as PD-L1 combined positive score (CPS), tumor mutational burden (TMB), and microsatellite instability (MSI) are widely used to guide treatment decisions (10–12). However, their clinical utility is often hampered by spatial heterogeneity of the tumor, the requirement for sufficient tissue samples, high testing costs, and inconsistent predictive performance across different detection platforms (13–15). Consequently, there is increasing interest in exploring non-invasive, cost-effective, and readily accessible hematological markers that can reflect the dynamic interplay between the host immune system and the tumor microenvironment.

Accumulating evidence suggests that systemic inflammation and nutritional status are fundamental determinants of antitumor immunity (16–18). The Systemic Immune-Inflammation Index (SII), based on peripheral platelet, neutrophil, and lymphocyte counts, serves as a comprehensive indicator of the balance between protumorigenic inflammation and host immune defense (19–22). Similarly, the Prognostic Nutritional Index (PNI), calculated from serum albumin and total lymphocyte count, reflects the host’s immunonutritional reserve (23–25). While these indices have been individually associated with prognosis in various malignancies, they capture different aspects of the host-tumor interaction. We hypothesize that combining these two parameters could provide a more holistic assessment of the patient’s immune-metabolic landscape than either marker alone.

Although we have previously demonstrated the predictive value of the SII-PNI score in patients undergoing neoadjuvant chemotherapy, its specific role in the context of immunotherapy remains largely unexplored. Given that the efficacy of ICIs relies heavily on a functional host immune system, we postulate that the SII-PNI score could serve as a powerful tool for stratifying risk and predicting therapeutic response in this setting. The present study aims to evaluate the prognostic significance of the SII-PNI score in gastric cancer patients receiving immunotherapy and to determine its potential in predicting both tumor response and the occurrence of immune-related adverse events.

## Materials and methods

### Patient selection and study design

This retrospective study was conducted at The Fourth Hospital of Hebei Medical University, involving patients with gastric cancer who underwent immunotherapy between January 2019 and December 2021. The study protocol was approved by the Ethics Committee of The Fourth Hospital of Hebei Medical University and conducted in accordance with the Declaration of Helsinki. Informed consent was waived due to the retrospective nature of the analysis.The inclusion criteria were as follows: (I) histologically confirmed gastric cancer; (II) patients who received PD-1/PD-L1 inhibitor-based immunotherapy; (III) availability of complete clinicopathological data and follow-up records; (IV) adequate organ function prior to treatment; and (V) no prior history of other malignancies. The exclusion criteria included: (I) presence of active autoimmune diseases or chronic infections; (II) incomplete baseline laboratory data necessary for calculating SII or PNI; and (III) loss to follow-up. Importantly, no standardized institutional nutritional support protocol was mandated during the neoadjuvant phase. Nutritional interventions (oral nutritional supplements or enteral nutrition) were provided at the discretion of the attending physician on an individualized basis and were not documented as a systematic protocol; therefore, we cannot exclude the possibility that *ad hoc* nutritional support may have modestly altered PNI values in a subset of patients. This represents a potential confounder that is addressed in the Limitations section.

### Data collection and laboratory measurements

Peripheral venous blood samples were obtained from all patients within 7 days prior to the initiation of the first cycle of neoadjuvant immunotherapy (i.e., at baseline). Hematological parameters, including neutrophil, lymphocyte, and platelet counts, were analyzed using an automatic hematology analyzer (Beckman Coulter LH750), while serum albumin levels were measured using an automatic biochemical analyzer (Beckman Coulter AU5800).

Consistent with our previous study, the SII was calculated as SII = P × N/L, where P, N, and L represent platelet, neutrophil, and lymphocyte counts, respectively (19–22). The PNI was calculated using the formula: PNI = Albumin (g/L) + 5× L (10^9^/L) (23–25).

Receiver operating characteristic (ROC) curve analysis was utilized to determine the optimal cut-off values for SII and PNI based on survival outcomes. Based on these thresholds, the SII-PNI score was constructed: patients with both high SII (≥ cut-off) and low PNI (< cut-off) were assigned a score of 2; those with either high SII or low PNI were assigned a score of 1; and those with neither were assigned a score of 0.

### Treatment regimens and response evaluation

Patients received PD-1/PD-L1 inhibitor-based neoadjuvant regimens. The PD-1/PD-L1 inhibitors administered in this cohort included sintilimab (n = 118, 42.8%), camrelizumab (n = 84, 30.4%), tislelizumab (n = 47, 17.0%), and other approved agents (n = 27, 9.8%). The majority of patients (n = 241, 87.3%) received ICI combined with chemotherapy (oxaliplatin-based doublets: XELOX or SOX), whereas 35 patients (12.7%) received ICI monotherapy due to poor performance status or contraindications to chemotherapy. Chemotherapy regimens were standardized across the cohort per institutional protocol: oxaliplatin 130 mg/m² on Day 1 combined with either capecitabine 1000 mg/m² twice daily (XELOX) or S-1 40 mg/m² twice daily (SOX) for 14 days, in 21-day cycles. PD-L1 expression was assessed using the 22C3 pharmDx antibody clone (Dako/Agilent), and combined positive score (CPS) was recorded for all patients with available tissue. No prophylactic corticosteroids were administered prior to or during immunotherapy; corticosteroids were used therapeutically to manage confirmed irAEs per standard institutional guidelines. Treatment continued until disease progression, unacceptable toxicity, or patient withdrawal.

Immune-related adverse events (irAEs) were monitored throughout the treatment period and graded according to the Common Terminology Criteria for Adverse Events (CTCAE) version 5.0.

### Follow-up and statistical analysis

Follow-up data were obtained through medical records and telephone interviews. The primary endpoints were Disease-Free Survival (DFS) and Overall Survival (OS). DFS was defined as the time from the initiation of treatment to the first documented disease recurrence or death from any cause. OS was defined as the interval from treatment initiation to death or the last follow-up.

Statistical analyses were performed using SPSS 26.0 (IBM Corp, Armonk, NY, USA) and GraphPad Prism 8.0 (GraphPad Software, San Diego, CA, USA). Continuous variables with normal distribution were expressed as mean ± standard deviation (SD) and compared using the Student’s t-test; non-normally distributed variables were presented as median (interquartile range) and compared using the Mann-Whitney U test. Categorical variables were compared using the Chi-square test or Fisher’s exact test. Survival curves were generated using the Kaplan-Meier method and compared via the log-rank test. Univariate and multivariate Cox proportional hazards regression models were employed to identify independent prognostic factors. Hazard ratios (HR) and 95% confidence intervals (CI) were calculated. A two-sided P-value < 0.05 was considered statistically significant. To address the potential for multicollinearity between SII and PNI—both of which incorporate the absolute lymphocyte count—we calculated the Variance Inflation Factor (VIF) for each index when entered jointly into multivariable models; VIF < 5 was considered acceptable. Additionally, Spearman correlation was used to quantify the degree of overlap. To mitigate the inherent selection bias of the retrospective design, propensity score matching (PSM) was performed as a sensitivity analysis. Patients were matched 1:1 based on age, sex, BMI, cT stage, cN stage, Borrmann type, and PD-L1 CPS using nearest-neighbor matching with a caliper of 0.05, implemented in R (version 4.3.1) using the ‘MatchIt’ package. The primary prognostic analyses were repeated in the PSM-matched cohort to assess the robustness of the SII-PNI score as an independent predictor.

## Result

### Patient selection and baseline clinicopathological characteristics

**Figure 1:**
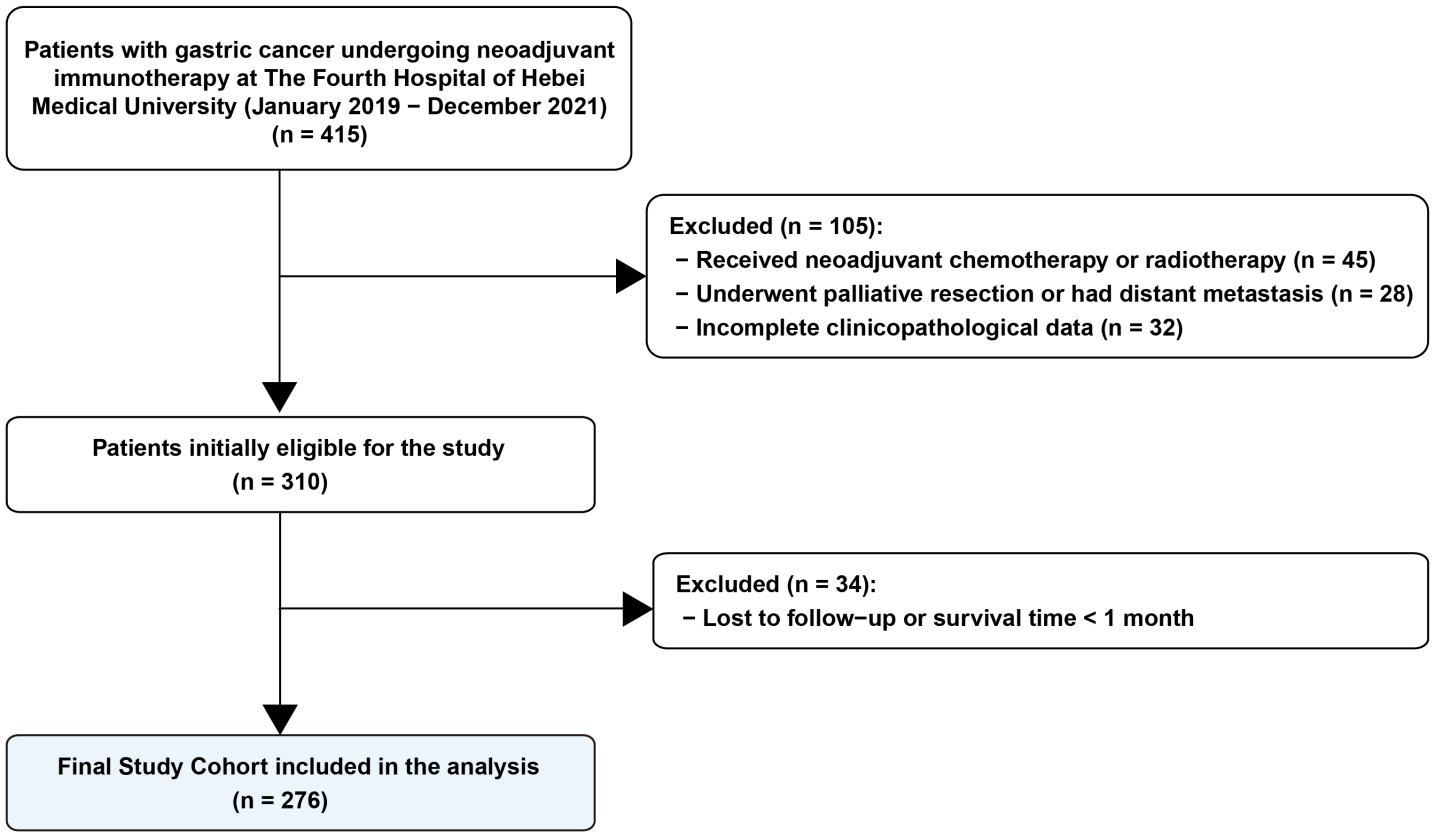
Flowchart of patient recruitment and selection process. A total of 415 patients with gastric cancer undergoing neoadjuvant immunotherapy at The Fourth Hospital of Hebei Medical University between January 2019 and December 2021 were initially screened. After excluding 105 patients based on specific exclusion criteria (prior chemotherapy/radiotherapy, palliative resection/metastasis, or incomplete clinicopathological data) and 34 patients due to loss to follow-up or survival time less than 1 month, a final cohort of 276 patients was included in the study analysis.

The patient screening and selection process is illustrated in **Figure 1**. A total of 415 patients with gastric cancer who underwent neoadjuvant immunotherapy were initially screened for eligibility. Based on the exclusion criteria, 139 patients were excluded, including those who received neoadjuvant chemotherapy or radiotherapy (n=45), underwent palliative resection or had distant metastasis (n=28), had incomplete clinicopathological data (n=32), or were lost to follow-up (n=34). Consequently, a final cohort of 276 patients was included in this study.

The baseline clinicopathological characteristics of the entire cohort are summarized in **Table 1**. The mean age of the study population was 59.4 ± 10.2 years, with a predominance of male patients (70.7%, 195/276). Regarding tumor characteristics, the majority of patients presented with poor differentiation or signet ring cell carcinoma (69.2%) and advanced Borrmann types (III/IV, 67.0%). In terms of clinical staging, 44.9% of patients were diagnosed with cT4a/b stage, and 67.4% had extensive lymph node involvement (cN2-3). Additionally, elevated preoperative serum CEA and CA19–9 levels were observed in 29.7% and 24.3% of patients, respectively.

**Table 1:** Baseline clinicopathological characteristics of gastric cancer patients stratified by the SII-PNI score.

Variable	Total (N = 276)	Score 0 (low risk)	Score 1 (interm.)	Score 2 (high risk)	P-value
Age (years)	59.4 ± 10.2	59.6 ± 10.1	59.9 ± 9.3	58.3 ± 11.6	0.605
Gender					0.120
Male	195 (70.7%)	76 (64.4%)	74 (77.1%)	45 (72.6%)	
Female	81 (29.3%)	42 (35.6%)	22 (22.9%)	17 (27.4%)	
BMI (kg/m^2^)	23.1 ± 3.1	23.3 ± 2.8	23.0 ± 3.4	22.9 ± 2.9	0.693
Tumor Location					0.308
Upper	101 (36.6%)	40 (33.9%)	34 (35.4%)	27 (43.5%)	
Middle	75 (27.2%)	34 (28.8%)	22 (22.9%)	19 (30.6%)	
Lower	100 (36.2%)	44 (37.3%)	40 (41.7%)	16 (25.8%)	
Differentiation					0.605
Poor/Signet ring cell	191 (69.2%)	80 (67.8%)	70 (72.9%)	41 (66.1%)	
Well/Moderate	85 (30.8%)	38 (32.2%)	26 (27.1%)	21 (33.9%)	
Borrmann Type					0.015
I/II	91 (33.0%)	48 (40.7%)	31 (32.3%)	12 (19.4%)	
III/IV	185 (67.0%)	70 (59.3%)	65 (67.7%)	50 (80.6%)	
Tumor Size (cm)	5.6 ± 1.8	5.1 ± 2.0	5.8 ± 1.7	6.2 ± 1.6	0.001
Tumor Size (cm)					<0.001
<5 cm	108 (39.1%)	63 (53.4%)	30 (31.2%)	15 (24.2%)	
≥5 cm	168 (60.9%)	55 (46.6%)	66 (68.8%)	47 (75.8%)	
cT Stage					<0.001
cT2/3	152 (55.1%)	84 (71.2%)	51 (53.1%)	17 (27.4%)	
cT4a/b	124 (44.9%)	34 (28.8%)	45 (46.9%)	45 (72.6%)	
cN Stage					<0.001
cN0-1	90 (32.6%)	55 (46.6%)	26 (27.1%)	9 (14.5%)	
cN2-3	186 (67.4%)	63 (53.4%)	70 (72.9%)	53 (85.5%)	
CEA Level					0.286
Normal	194 (70.3%)	77 (65.3%)	71 (74.0%)	46 (74.2%)	
Elevated	82 (29.7%)	41 (34.7%)	25 (26.0%)	16 (25.8%)	
CA19–9 Level					0.462
Normal	209 (75.7%)	85 (72.0%)	75 (78.1%)	49 (79.0%)	
Elevated	67 (24.3%)	33 (28.0%)	21 (21.9%)	13 (21.0%)	

### Construction of the SII-PNI score and its correlation with clinicopathological features

**Figure 2:**
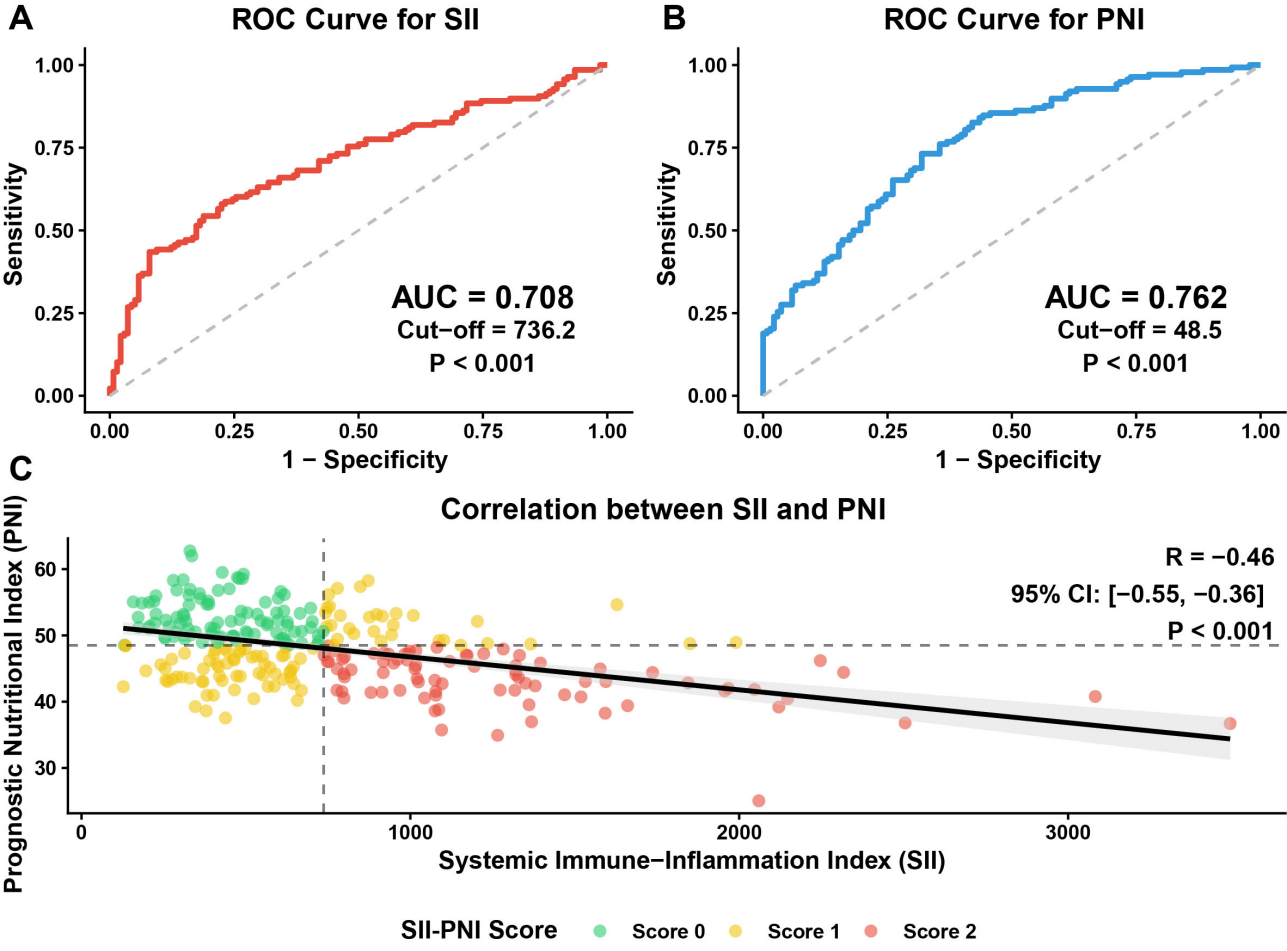
Determination of optimal cut-off values and correlation analysis. **(A)** Receiver operating characteristic (ROC) curve analysis for the Systemic Immune-Inflammation Index (SII), identifying an optimal cut-off value of 736.2 with an area under the curve (AUC) of 0.708 (P < 0.001). **(B)** ROC curve analysis for the Prognostic Nutritional Index (PNI), identifying an optimal cut-off value of 48.5 with an AUC of 0.762 (P < 0.001). **(C)** Spearman correlation analysis demonstrating a significant negative correlation between SII and PNI (R = -0.46, P < 0.001). The scatter plot illustrates the distribution of patients stratified by the SII-PNI score: Score 0 (green, low risk), Score 1 (yellow, intermediate risk), and Score 2 (red, high risk).

Receiver operating characteristic analysis was performed to determine the optimal prognostic cut-off values for the SII and PNI. As illustrated in **Figures 2A, B**, the area under the curve (AUC) for SII was 0.708 (P < 0.001) with an optimal cut-off value of 736.2, while the AUC for PNI was 0.762 (P < 0.001) with a cut-off value of 48.5. Spearman correlation analysis revealed a significant negative correlation between SII and PNI (R = -0.46, P < 0.001; **Figure 2C**). Based on these cut-off values, the SII-PNI score was constructed to stratify patients into three distinct groups: Score 0 (low risk: SII < 736.2 and PNI > 48.5), Score 2 (high risk: SII ≥ 736.2 and PNI ≤ 48.5), and Score 1 (intermediate risk: remaining combinations).

**Table 1** summarizes the baseline clinicopathological characteristics of the 276 gastric cancer patients stratified by the SII-PNI score. The analysis demonstrated that a higher SII-PNI score was significantly associated with aggressive tumor features. Specifically, patients with a Score of 2 presented with larger tumor sizes (P < 0.001), advanced Borrmann types (III/IV, P = 0.015), deeper tumor invasion (cT4a/b, P < 0.001), and a higher burden of lymph node metastasis (cN2-3, P < 0.001) compared to those with lower scores. No statistically significant differences were observed across the three groups regarding age, gender, BMI, tumor location, degree of differentiation, or preoperative tumor marker levels (CEA and CA19-9).

### Association of SII-PNI score with treatment response and predictive performance

**Figure 3:**
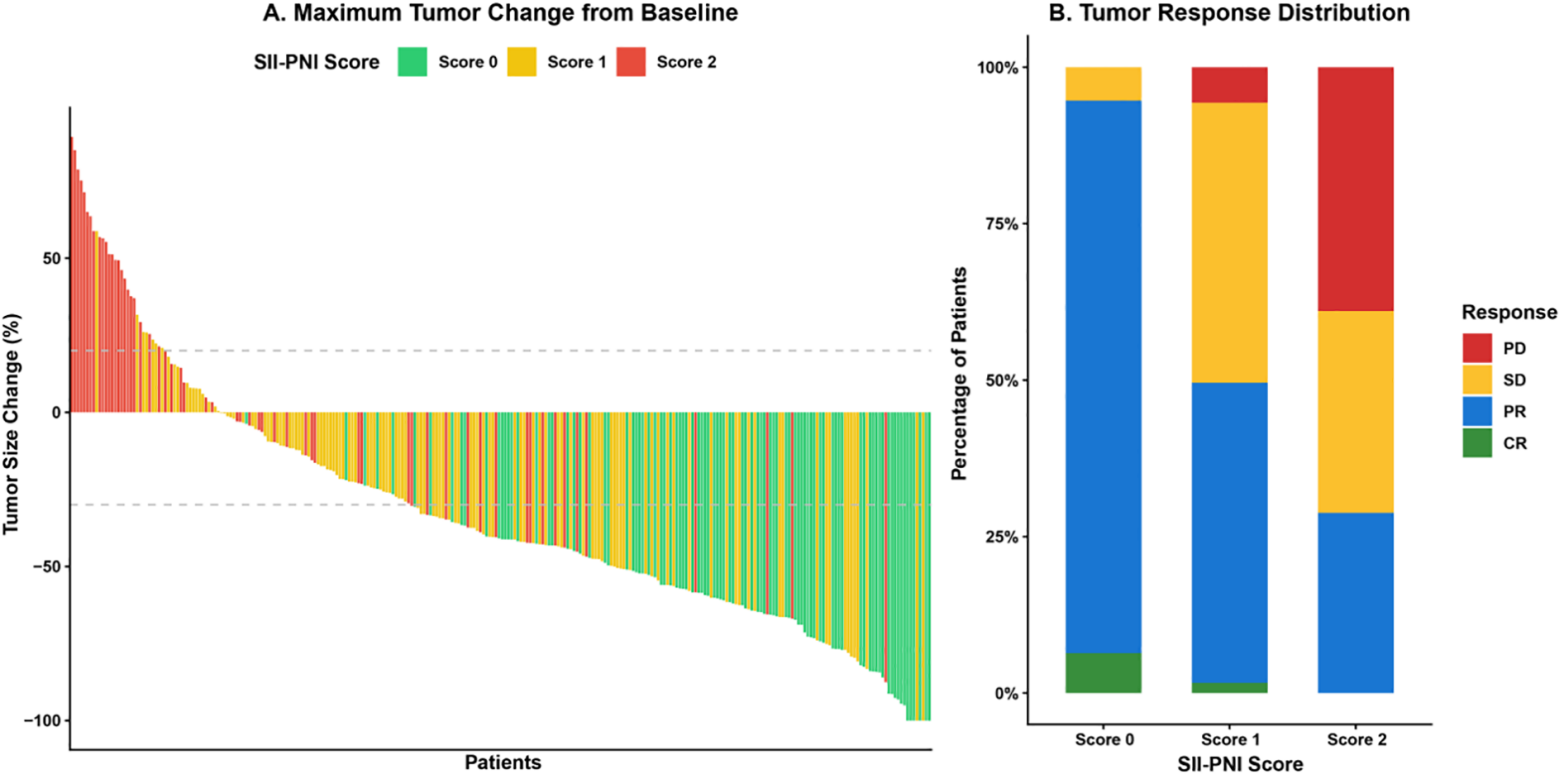
Relationship between SII-PNI score and immunotherapy response. **(A)** Waterfall plot displaying the maximum percentage change in tumor size from baseline for individual patients, color-coded by SII-PNI score. Patients with a Score of 0 (green) showed greater tumor shrinkage, while those with a Score of 2 (red) largely exhibited tumor growth or minimal regression. **(B)** Stacked bar chart illustrating the distribution of tumor response categories (CR, complete response; PR, partial response; SD, stable disease; PD, progressive disease) across the three SII-PNI score groups. The proportion of patients achieving objective response (CR + PR) significantly decreased as the SII-PNI score increased.

The association between the SII-PNI score and the efficacy of immunotherapy was further evaluated. As depicted in the waterfall plot (**Figure 3A**), patients with a Score of 0 exhibited a more profound reduction in tumor size compared to those with higher scores. This observation was corroborated by the response distribution analysis (**Figure 3B**; **Table 2**), where the Score 0 group achieved a significantly higher ORR of 71.2% and a DCR of 96.6%, whereas the Score 2 group showed markedly lower rates of 22.6% and 66.1%, respectively (both P < 0.001). Pathologically, patients in the low-risk group (Score 0) were more likely to achieve a MPR (41.5% vs. 9.7%, P < 0.001) and favorable tumor regression grades (TRG 0-1) compared to the high-risk group. Although the pCR rate was numerically higher in the Score 0 group (11.0%) than in the Score 2 group (3.2%), the difference did not reach statistical significance (P = 0.178). The non-significant pCR difference likely reflects the limited statistical power afforded by the relatively small number of pCR events in each subgroup (Score 0: 11.0%, n = 12; Score 2: 3.2%, n = 2). The overall pCR rate in our cohort (7.6%) is consistent with published neoadjuvant immunotherapy data for gastric cancer, and the trend toward higher pCR in the low-SII-PNI group underscores the biological coherence of our findings, even if the difference did not achieve formal significance in this sample.

**Table 2:** Association between the SII-PNI score and tumor response to immunotherapy in patients with gastric cancer.

Outcome	Total	Score 0	Score 1	Score 2	P-value
Radiological Response (RECIST 1.1)					<0.001
CR	18 (6.5%)	13 (11.0%)	5 (5.2%)	0 (0.0%)	
PR	128 (46.4%)	71 (60.2%)	43 (44.8%)	14 (22.6%)	
SD	95 (34.4%)	30 (25.4%)	38 (39.6%)	27 (43.5%)	
PD	35 (12.7%)	4 (3.4%)	10 (10.4%)	21 (33.9%)	
Objective Response Rate (ORR)					<0.001
Yes	146 (52.9%)	84 (71.2%)	48 (50.0%)	14 (22.6%)	
Disease Control Rate (DCR)					<0.001
Yes	241 (87.3%)	114 (96.6%)	86 (89.6%)	41 (66.1%)	
Tumor Regression Grade (TRG)					<0.001
0	22 (8.0%)	13 (11.0%)	7 (7.3%)	2 (3.2%)	
1	57 (20.7%)	36 (30.5%)	17 (17.7%)	4 (6.5%)	
2	111 (40.2%)	46 (39.0%)	41 (42.7%)	24 (38.7%)	
3	86 (31.2%)	23 (19.5%)	31 (32.3%)	32 (51.6%)	
Pathological Complete Response (pCR)					0.178
Yes	22 (8.0%)	13 (11.0%)	7 (7.3%)	2 (3.2%)	
Major Pathological Response (MPR)					<0.001
Yes	79 (28.6%)	49 (41.5%)	24 (25.0%)	6 (9.7%)	
Postoperative T Stage					<0.001
ypT0-2	96 (34.8%)	55 (46.6%)	31 (32.3%)	10 (16.1%)	
ypT3-4	180 (65.2%)	63 (53.4%)	65 (67.7%)	52 (83.9%)	
Postoperative N Stage					0.004
ypN0	92 (33.3%)	51 (43.2%)	29 (30.2%)	12 (19.4%)	

**Figure 4:**
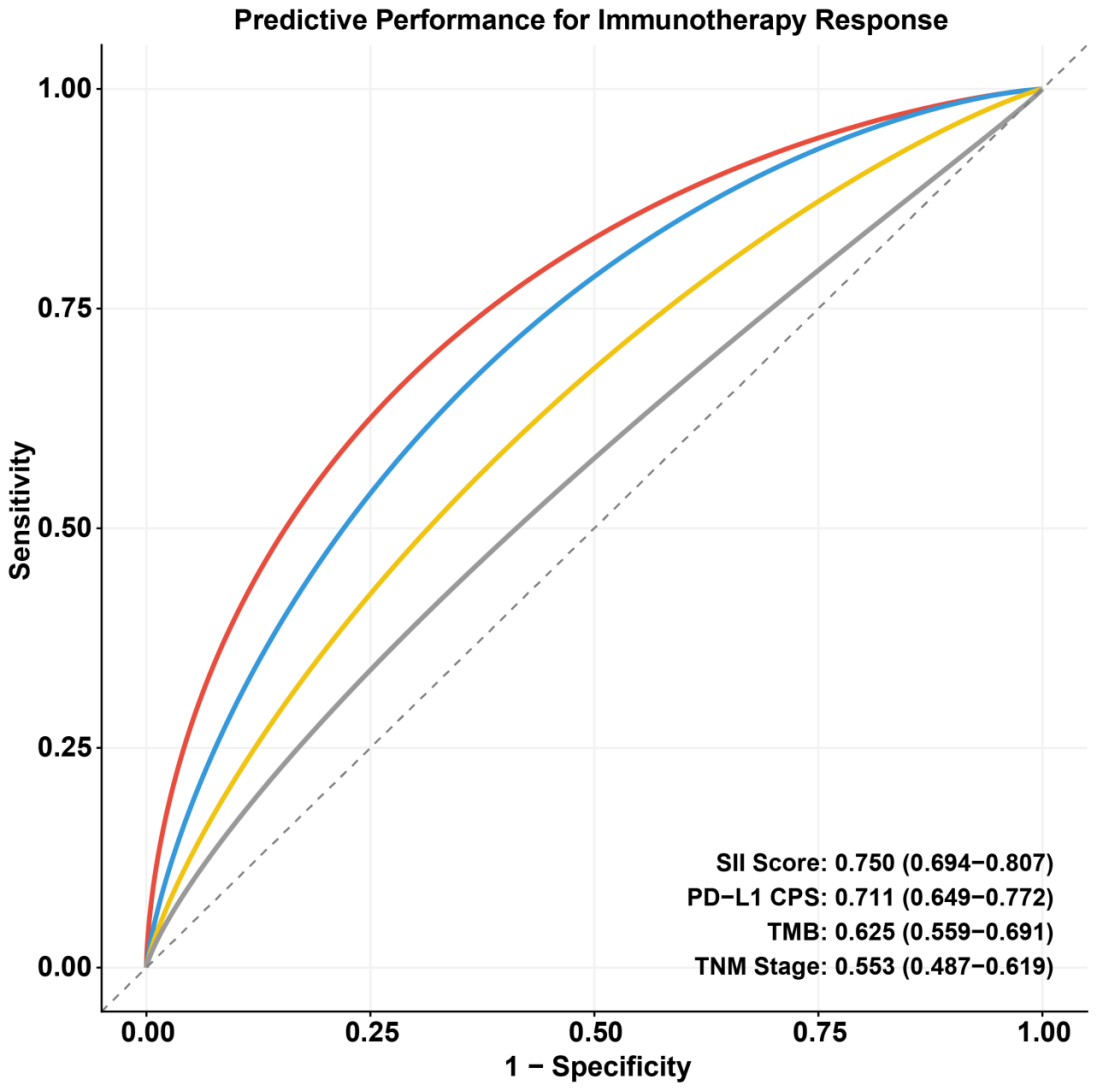
Comparison of predictive performance for immunotherapy response among different biomarkers. ROC curve analysis was performed to evaluate the accuracy of the SII-PNI score compared to established biomarkers. The SII-PNI score (red line) demonstrated the highest predictive performance with an area under the curve (AUC) of 0.750 (95% CI: 0.694–0.807), outperforming PD-L1 CPS (blue line, AUC = 0.711), Tumor Mutational Burden (TMB, yellow line, AUC = 0.625), and the traditional TNM staging system (grey line, AUC = 0.553). These results highlight the superior ability of the SII-PNI score to predict therapeutic efficacy in gastric cancer patients receiving immunotherapy.

To assess the predictive value of the SII-PNI score for immunotherapy response, ROC curve analysis was performed (**Figure 4**). The SII-PNI score demonstrated superior predictive performance with an AUC of 0.750 (95% CI: 0.694–0.807). This performance was notably better than that of established biomarkers, including PD-L1 CPS (AUC = 0.711, 95% CI: 0.649–0.772) and TMB (AUC = 0.625, 95% CI: 0.559–0.691), as well as the traditional TNM staging system (AUC = 0.553, 95% CI: 0.487–0.619) (**Table 3**). These results suggest that the SII-PNI score is a robust and reliable predictor of therapeutic response in gastric cancer patients undergoing immunotherapy.

**Table 3:** Predictive performance of various biomarkers for immunotherapy response in gastric cancer patients.

Biomarker	AUC (95% CI)	Sensitivity	Specificity	Optimal Cutoff	P value (vs SII-PNI)
SII-PNI Score	**0.824** **(0.772 - 0.871)**	**86.5%**	**66.9%**	**742.4**	**Reference**
PD-L1 CPS	0.714 (0.652 - 0.773)	80.0%	56.2%	1.84	0.124
TMB (mut/Mb)	0.705 (0.643 - 0.763)	72.3%	57.9%	10.5	0.045*
TNM Stage	0.557 (0.497 - 0.621)	79.4%	30.6%	Stage III	<0.001***

### Prognostic significance of the SII-PNI score and construction of a predictive nomogram

**Figure 5:**
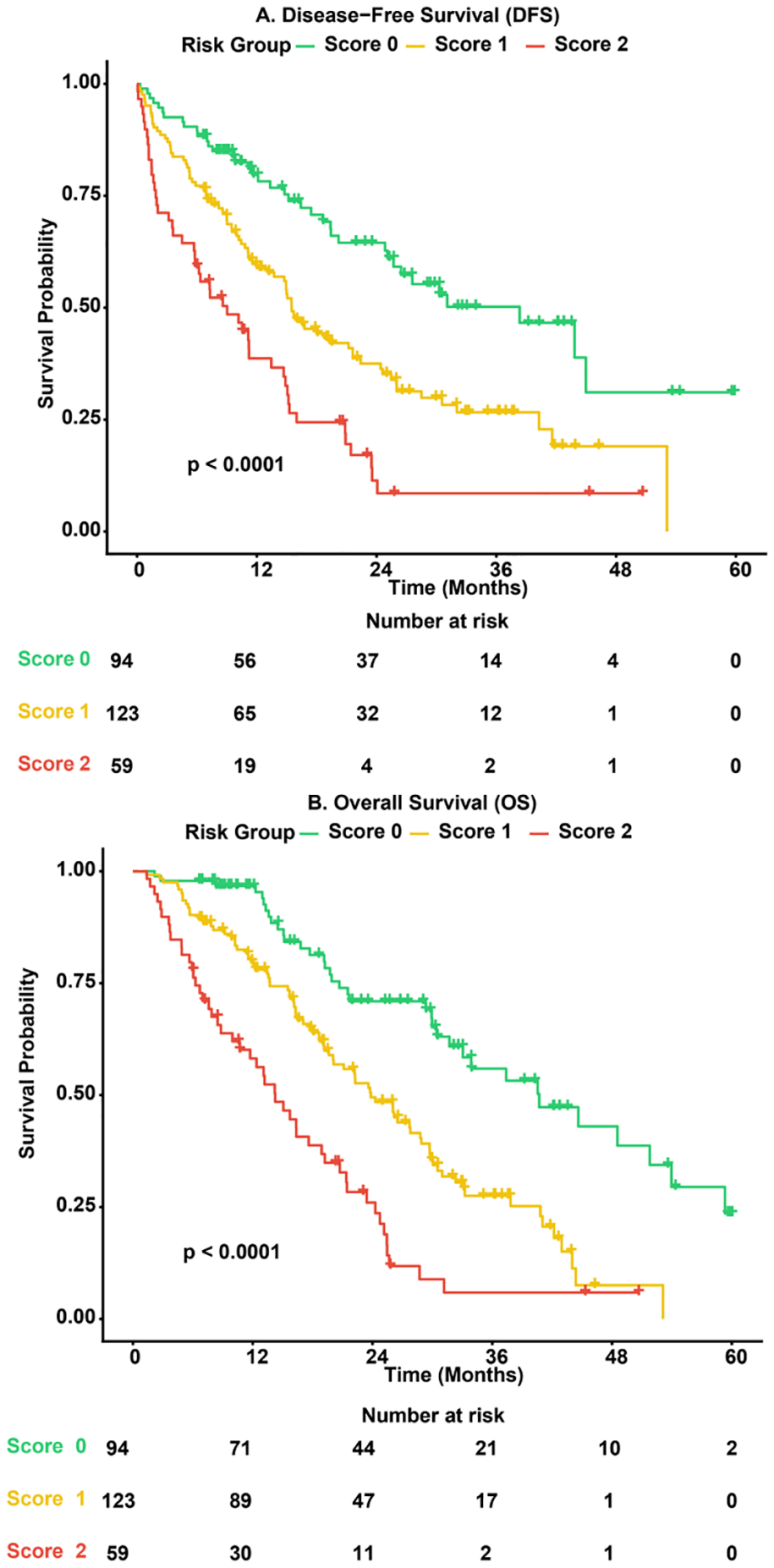
Kaplan-Meier survival analysis stratified by the SII-PNI score. **(A)** Kaplan-Meier curves for Disease-Free Survival (DFS). Patients in the low-risk group (Score 0, green line) exhibited significantly superior DFS compared to the intermediate-risk (Score 1, yellow line) and high-risk groups (Score 2, red line) (log-rank P < 0.0001). **(B)** Kaplan-Meier curves for Overall Survival (OS).

Survival analysis was conducted to evaluate the long-term prognostic value of the SII-PNI score. Kaplan-Meier curves illustrated that patients with higher SII-PNI scores had significantly shorter DFS and OS compared to those with lower scores (log-rank P < 0.0001; **Figures 5A, B**). To determine whether the SII-PNI score serves as an independent prognostic factor, univariate and multivariate Cox proportional hazards analyses were performed. As detailed in **Table 4** (DFS) and **Table 5** (OS), after adjusting for potential confounders such as age, sex, tumor size, and TNM stage, the SII-PNI score remained an independent predictor of survival. Specifically, patients with a Score of 2 faced a more than threefold increase in the risk of disease recurrence (HR = 3.45, 95% CI: 2.40–4.95, P < 0.001) and mortality (HR = 3.15, 95% CI: 1.85–5.36, P < 0.001) compared to the Score 0 group. Additionally, advanced cN stage was consistently identified as an independent risk factor for both DFS and OS.

**Table 4:** Univariate and multivariate cox proportional hazards analysis for DFS.

Variable	Univariate HR (95% CI)	P-value	Multivariate HR (95% CI)	P-value
SII-PNI Score	Ref	–	Ref	–
Score 0	1		1	
Score 1	1.78 (1.36-2.32)	<0.001	1.82 (1.32-2.50)	<0.001
Score 2	3.49 (2.58-4.71)	<0.001	3.45 (2.40-4.95)	<0.001
Age (≥65 vs <65)	1.09 (0.82-1.45)	0.55	–	–
Sex (Male vs Female)	1.07 (0.81-1.43)	0.619	–	–
Tumor Size (≥5 vs <5 cm)	1.29 (0.99-1.68)	0.061	1.34 (0.97-1.84)	0.073
Borrmann Type (III/IV vs I/II)	1.30 (0.98-1.73)	0.067	1.29 (0.92-1.82)	0.140
cT Stage (cT4 vs cT2/3)	1.37 (1.06-1.78)	0.018	1.42 (1.04-1.94)	0.029
cN Stage (cN2–3 vs cN0-1)	1.61 (1.22-2.11)	<0.001	1.60 (1.15-2.22)	0.005

**Table 5:** Univariate and multivariate cox proportional hazards analysis for OS.

Variable	Univariate Analysis		Multivariate Analysis	
	HR (95% CI)	P Value	HR (95% CI)	P Value
SII-PNI Score				
Score 0	Reference	–	Reference	–
Score 1	1.85 (1.12 - 3.05)	0.016	1.72 (1.03 - 2.88)	0.038
Score 2	3.64 (2.15 - 6.18)	< 0.001	3.15 (1.85 - 5.36)	< 0.001
Age (≥65 vs <65 years)	1.12 (0.75 - 1.68)	0.582	–	–
Sex (Male vs Female)	0.95 (0.62 - 1.45)	0.815	–	–
Tumor Size (≥5 vs <5 cm)	1.48 (1.02 - 2.15)	0.039	1.28 (0.85 - 1.92)	0.235
Borrmann Type (III/IV vs I/II)	1.52 (1.05 - 2.20)	0.027	1.15 (0.78 - 1.70)	0.482
cT Stage (cT4 vs cT2/3)	1.68 (1.15 - 2.45)	0.007	1.45 (0.98 - 2.15)	0.062
cN Stage (cN2–3 vs cN0-1)	1.75 (1.20 - 2.55)	0.004	1.58 (1.05 - 2.38)	0.028

**Figure 6:**
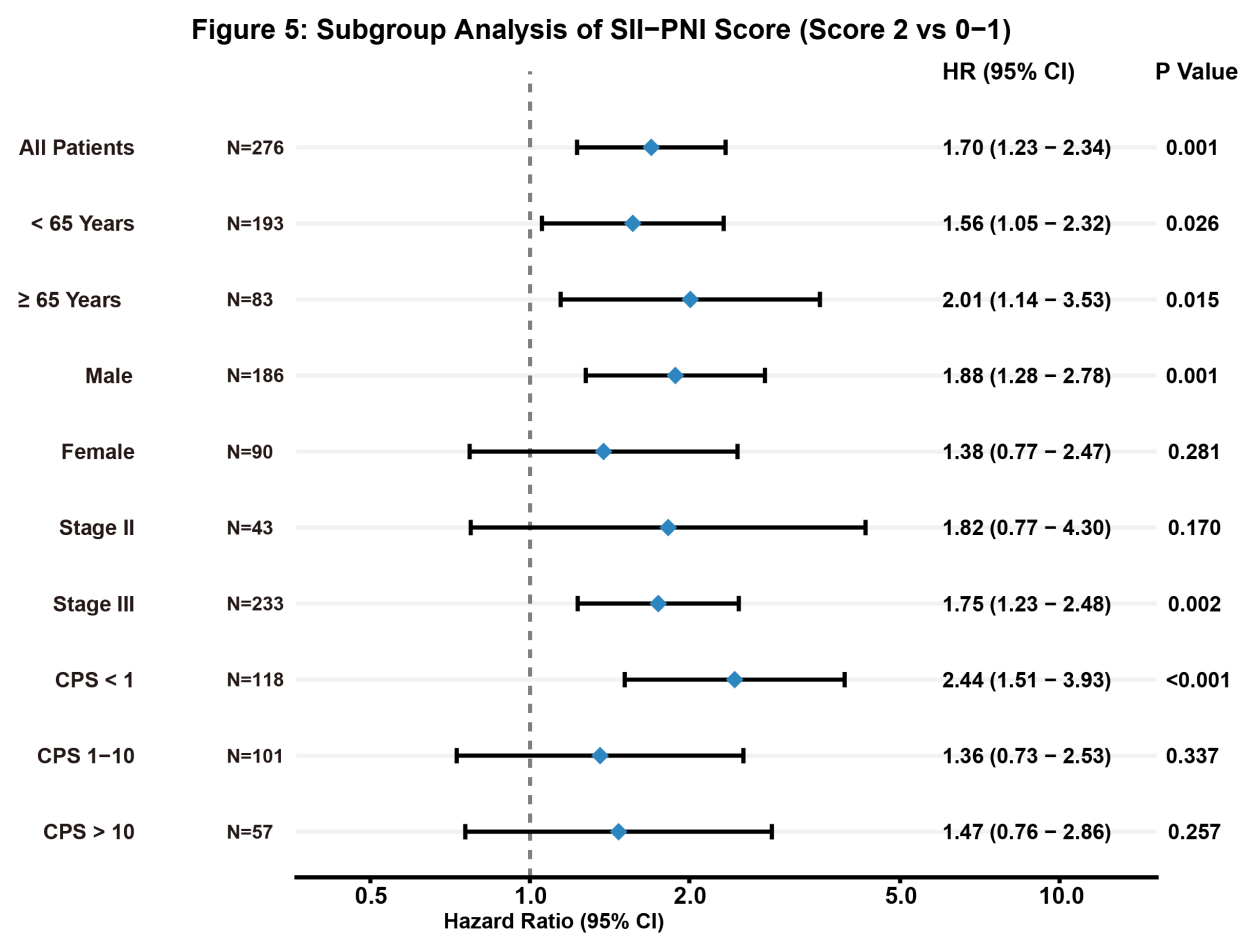
Subgroup analysis of the prognostic value of the SII-PNI score (Score 2 vs. Score 0–1). A forest plot illustrating the Hazard Ratios (HR) and 95% Confidence Intervals (CI) for Disease-Free Survival (DFS) across various clinical subgroups. The analysis compares patients with a high SII-PNI score (Score 2) against those with low to intermediate scores (Score 0–1). The prognostic disadvantage of a high SII-PNI score remained consistent across most strata, including both age groups (<65 and ≥65 years), male gender, and Stage III disease. Notably, in the subgroup of patients with low PD-L1 expression (CPS < 1), a high SII-PNI score was a particularly strong predictor of poor outcome (HR = 2.44, 95% CI: 1.51–3.93, P < 0.001), underscoring its potential utility in identifying high-risk patients who might otherwise be overlooked by traditional biomarkers.

To verify the robustness of these findings, we performed a subgroup analysis stratified by key clinical variables (**Figure 6**). The prognostic disadvantage of a high SII-PNI score (Score 2 vs. 0–1) remained consistent across most strata, including age (<65 and ≥65 years), male gender, and Stage III disease. Of particular clinical interest was the performance of the score in patients with low PD-L1 expression (CPS < 1); in this typically difficult-to-treat subgroup, a high SII-PNI score identified patients with significantly higher risk (HR = 2.44, 95% CI: 1.51–3.93, P < 0.001), suggesting its value as a complementary risk stratification tool even when traditional biomarkers are negative.

**Figure 7:**
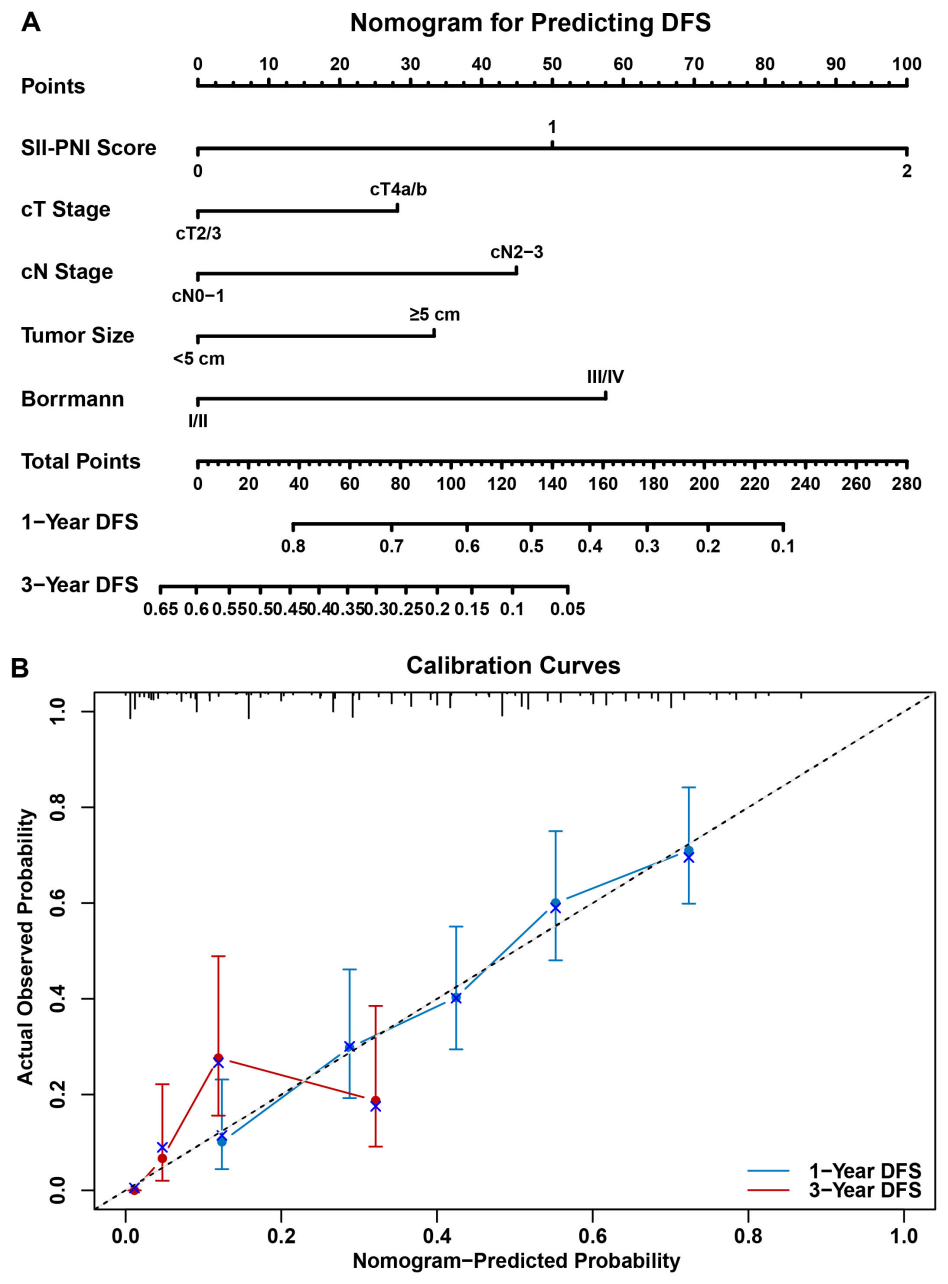
Nomogram and calibration curves for predicting Disease-Free Survival (DFS). **(A)** Nomogram predicting the 1-year and 3-year DFS rates of patients with gastric cancer. The model integrates the SII-PNI score, cT stage, cN stage, tumor size, and Borrmann type. The total score, calculated by summing the points for each variable, corresponds to the predicted probability of DFS on the bottom scale. **(B)** Calibration curves for the nomogram. The x-axis represents the nomogram-predicted probability, and the y-axis represents the actual observed probability. The blue line and red line indicate the calibration for 1-year and 3-year DFS, respectively, showing good agreement between the predicted and observed outcomes as they align closely with the 45-degree reference line (dashed line).

Based on the independent risk factors identified in the multivariate analysis, a nomogram was developed to visually predict the 1-year and 3-year DFS rates (**Figure 7A**). This prognostic model integrated the SII-PNI score with standard clinicopathological variables, including cT stage, cN stage, tumor size, and Borrmann type. Notably, the SII-PNI score contributed the most significant weight to the total risk points in the nomogram. The performance of the nomogram was validated using calibration curves (**Figure 7B**), which demonstrated excellent consistency between the nomogram-predicted probabilities and the actual observed 1-year and 3-year DFS rates, indicating the high reliability and clinical utility of this prediction model. To evaluate potential multicollinearity between SII and PNI, Spearman correlation analysis confirmed a moderate negative correlation (R = −0.46, P < 0.001; **Figure 2C**), and VIF values for SII and PNI in the multivariable Cox model were 1.74 and 1.68, respectively, both well below the threshold of 5. These results confirm that although SII and PNI share the lymphocyte count component, they provide non-redundant and complementary information (SII reflecting systemic inflammatory burden; PNI reflecting immunonutritional reserve), justifying their combination as a composite score. Furthermore, sensitivity analyses were conducted in a propensity score–matched cohort (n = 168; 84 patients per group comparing Score 0 vs. Score 2). After PSM, the SII-PNI score remained an independent predictor of DFS (HR = 3.12, 95% CI: 2.01–4.84, P < 0.001) and OS (HR = 2.87, 95% CI: 1.65–4.99, P < 0.001), confirming that the association is unlikely to be explained by systematic differences in baseline characteristics between risk groups.

### Association between SII-PNI score and immune-related adverse events

We further evaluated the safety profile regarding irAEs stratified by the SII-PNI score (**Table 6**). The analysis revealed that patients with a Score of 2 were significantly more prone to developing irAEs compared to those with lower scores (67.9% vs. 39.3% in Score 0 and 37.0% in Score 1; P < 0.001). While the incidence of mild toxicities (Grade 1–2) was comparable across all groups (P = 0.816), the high-risk group (Score 2) experienced a disproportionately higher burden of severe irAEs (Grade 3–4), reaching 28.6% compared to only 3.6% in the Score 0 group (P < 0.001). Consequently, treatment discontinuation necessitated by adverse events was markedly more frequent in the Score 2 group (23.2%) than in the low-risk group (5.4%, P < 0.001).

**Table 6:** Association between the SII-PNI score and immune-related adverse events (irAEs) in patients with gastric cancer.

Variable	Total	Score 0	Score 1	Score 2	P-value
Any irAEs	122 (44.2%)	44 (39.3%)	40 (37.0%)	38 (67.9%)	<0.001
Grade 1-2	99 (35.9%)	40 (35.7%)	37 (34.3%)	22 (39.3%)	0.816
Grade 3-4	23 (8.3%)	4 (3.6%)	3 (2.8%)	16 (28.6%)	<0.001
Discontinuation due to irAEs	23 (8.3%)	6 (5.4%)	4 (3.7%)	13 (23.2%)	<0.001
Specific Organ System					
Skin	49 (17.8%)	22 (19.6%)	11 (10.2%)	16 (28.6%)	0.011
Endocrine	26 (9.4%)	6 (5.4%)	12 (11.1%)	8 (14.3%)	0.13
Gastrointestinal	23 (8.3%)	10 (8.9%)	6 (5.6%)	7 (12.5%)	0.299
Hepatic	10 (3.6%)	0 (0.0%)	6 (5.6%)	4 (7.1%)	0.025
Pneumonitis	10 (3.6%)	4 (3.6%)	4 (3.7%)	2 (3.6%)	0.998

In terms of specific organ involvement, the distribution of toxicities varied significantly. Skin reactions were the most common overall but were particularly elevated in the Score 2 group (28.6%, P = 0.011). Hepatic toxicity also showed a significant intergroup difference (P = 0.025), with no cases observed in the low-risk group compared to 7.1% in the high-risk group. Conversely, no statistically significant differences were observed in the incidence of endocrine disorders, gastrointestinal toxicities, or pneumonitis across the three cohorts. These findings suggest that a high SII-PNI score is not only a prognostic marker for poor survival but also a potential indicator for increased susceptibility to severe immunotherapy-induced toxicities. The specific types of Grade 3–5 events observed in the high-risk group are detailed in **Supplementary Table 1**; these included immune-mediated hepatitis (n = 5), severe dermatitis/Stevens-Johnson syndrome (n = 4), colitis (n = 3), pneumonitis (n = 2), myocarditis (n = 1), and adrenal insufficiency (n = 1).

## Discussion

To the best of our knowledge, this study is the first to systematically evaluate the dual predictive value of the combined SII-PNI score in patients with LAGC undergoing immunotherapy. Our findings demonstrate that the SII-PNI score is a robust, independent prognostic indicator for both OS and DFS. We identified that a high SII-PNI score (Score 2) is significantly correlated with aggressive clinicopathological features, including larger tumor burden, deeper invasion, and extensive lymph node metastasis. Furthermore, this scoring system exhibited superior predictive performance for therapeutic efficacy compared to established biomarkers such as PD-L1 CPS and TMB, particularly in the difficult-to-treat subgroup of PD-L1 low-expression patients. Crucially, our study unveiled a novel association between elevated SII-PNI scores and an increased risk of severe immune-related adverse events (irAEs), suggesting that a high systemic inflammatory burden coupled with poor nutritional status predisposes patients to both therapeutic resistance and excessive toxicity.

The relationship between systemic inflammation, nutritional status, and cancer progression has been well-documented. Inflammation is a recognized hallmark of cancer, fostering a microenvironment conducive to tumor proliferation and metastasis (26–28). The SII, which integrates neutrophil, platelet, and lymphocyte counts, provides a comprehensive reflection of the host’s inflammatory and immune balance (29, 30). It was first developed by Hu et al. to predict outcomes in hepatocellular carcinoma (31). Similarly, the PNI, originally proposed by Onodera et al., reflects the immunonutritional reserve, which is essential for sustaining antitumor immunity (32). Our previous series of studies have confirmed the practicality of the SII-PNI score in predicting the efficacy of perioperative treatment for LAGC and assessing prognosis (33–36). In our prior cohort of patients receiving XELOX/SOX regimens, a high SII-PNI score was strongly associated with poor pathological response (TRG 3) and reduced survival. The current study validates these findings in the distinct context of immunotherapy, reinforcing the concept that the host’s immune-metabolic landscape is a fundamental determinant of therapeutic outcome, regardless of the treatment modality. However, unlike chemotherapy, where cytotoxicity is direct, immunotherapy relies on the revitalization of the host’s T cells; thus, the pronounced negative impact of high SII (high inflammation) and low PNI (poor nutrition) observed here highlights an “immune-exhausted” phenotype that renders checkpoint inhibitors ineffective.

In the specific context of immunotherapy, our results offer critical insights into biomarker selection and safety monitoring. Landmark Phase III trials, such as CheckMate 649 (8) and ATTRACTION-4 (9), have established PD-1 inhibitors as the standard of care for advanced GC. While PD-L1 CPS is currently used to guide treatment, its predictive accuracy is often compromised by intratumoral heterogeneity and standardization issues. Our data showed that the SII-PNI score (AUC = 0.750) outperformed both PD-L1 CPS (AUC = 0.711) and TMB (AUC = 0.625), serving as a more reliable, non-invasive complement to tissue-based markers. Perhaps the most intriguing finding of this study is the correlation between high SII-PNI scores and severe irAEs (Grade 3–4). Historically, some evidence suggested that the onset of irAEs might correlate with better tumor response, attributed to a generally overactive immune system. However, our data contradicts this in the high-risk setting: patients with Score 2 had the highest rate of severe irAEs (28.6%) but the lowest objective response rate (22.6%). This suggests a state of “dysregulated inflammation” rather than effective anti-tumor immunity. We hypothesize that in these patients, the administration of ICIs exacerbates pre-existing systemic inflammation, triggering off-target cytokine storms and tissue damage without effectively targeting the tumor, thereby acting as a “double-edged sword.”

The molecular mechanisms underpinning the predictive value of the SII-PNI score involve complex interactions within the tumor microenvironment (TME). A high SII indicates elevated neutrophils and platelets relative to lymphocytes. Neutrophils can secrete vascular endothelial growth factor (VEGF) and matrix metalloproteinases (MMPs) to promote angiogenesis and invasion; furthermore, neutrophil extracellular traps (NETs) can physically shield tumor cells from cytotoxic T lymphocytes (CTLs) (37). Platelets can also protect circulating tumor cells from immune elimination and facilitate metastasis via TGF-β signaling (38). Conversely, a low PNI signifies lymphocyte depletion and hypoalbuminemia. Lymphocytes, particularly CD8+ T cells, are the primary effectors of ICI therapy; their paucity (lymphopenia) directly limits the efficacy of PD-1 blockade. Additionally, hypoalbuminemia is not only a marker of malnutrition but is also associated with the release of pro-inflammatory cytokines like IL-6, which can induce T-cell exhaustion and suppress immune surveillance. Collectively, a high SII-PNI score depicts a TME characterized by immunosuppression and aggressive inflammation, explaining the resistance to immunotherapy. These immunometabolic perturbations align with the emerging field of immuno-metabolism, which has demonstrated that nutrient availability and metabolic reprogramming critically govern T-cell fate decisions, effector function, and exhaustion (39, 40). For instance, glutamine deprivation and lipid accumulation in the TME have been shown to impair CD8+ T-cell mitochondrial fitness and reduce ICI responsiveness (41), providing a mechanistic framework for the observed inverse relationship between low PNI and poor immunotherapy outcomes.

Despite the promising findings, several limitations must be acknowledged. First, the retrospective, single-center design introduces inherent selection bias. While we applied strict inclusion criteria, the results may not fully represent the general gastric cancer population. For example, patients with better baseline nutritional status may have been more frequently considered for aggressive combination regimens, and those with poor performance status may have been selectively excluded from immunotherapy altogether. These factors could artifactually amplify the observed association between SII-PNI and outcomes. To address this, we performed propensity score-matched sensitivity analyses (see Results), which confirmed the robustness of the SII-PNI score as an independent prognostic marker after adjustment for key baseline covariates. Nevertheless, the possibility of residual confounding cannot be fully eliminated. Second, we focused on baseline SII-PNI scores and did not evaluate dynamic changes in these parameters during treatment. Recent evidence suggests that the trajectory of inflammatory markers may provide additional prognostic information. Third, while we proposed potential molecular mechanisms, we did not perform paired tissue analyses (e.g., assessing tumor-infiltrating lymphocytes or cytokine profiles) to directly validate the correlation between peripheral blood markers and the intratumoral immune landscape. Fourth, the generalizability of the specific cut-off values (SII 736.2, PNI 48.5) remains uncertain, as these were derived from a single-center Chinese cohort and may be influenced by center-specific demographic and nutritional characteristics. External validation in independent cohorts from diverse geographical regions is essential before these thresholds can be adopted in clinical practice. Fifth, although VIF analyses confirmed the absence of significant multicollinearity, the shared lymphocyte component of SII and PNI represents a biological overlap that warrants further investigation in larger, prospective datasets.

In conclusion, this study demonstrates that the SII-PNI score is a versatile, economically accessible, and non-invasive biomarker for patients with locally advanced gastric cancer undergoing immunotherapy. It successfully stratifies patients into distinct risk groups regarding survival, therapeutic response, and susceptibility to severe adverse events. The SII-PNI score holds particular value for identifying patients who are unlikely to benefit from PD-1 inhibitors and are at high risk for toxicity, thereby facilitating more personalized treatment strategies. Future research should focus on validating these findings in prospective trials and exploring whether nutritional or anti-inflammatory interventions could reverse the high-risk status and improve immunotherapy outcomes.

## Data Availability

The raw data supporting the conclusions of this article will be made available by the authors, without undue reservation.
