# Supplementary material for: An integrated SII-PNI immune-nutritional scoring system predicts efficacy and immune-related adverse events in locally advanced gastric cancer patients undergoing neoadjuvant immunotherapy
Source: Front Immunol. 2026 Apr 30;17:1806537. doi: 10.3389/fimmu.2026.1806537 (PMC13171329; doi:10.3389/fimmu.2026.1806537)
Supplement: Supplementary file 1 [file Table1.docx]

**Supplementary Table S1. Detailed composition of severe immune-related adverse events in high-risk patients (SII-PNI Score 2)**

| **Event category** | **Specific severe irAE** | **Organ system** | **No. of patients, n** | **% of Score 2 patients (n=56)** | **% of severe irAEs (n=16)** |
| --- | --- | --- | --- | --- | --- |
| Immune-mediated hepatitis | Immune-mediated hepatitis | Hepatic | 5 | 8.9 | 31.3 |
| Severe dermatologic toxicity | Severe dermatitis/Stevens–Johnson syndrome | Skin | 4 | 7.1 | 25.0 |
| Immune-mediated colitis | Colitis | Gastrointestinal | 3 | 5.4 | 18.8 |
| Immune-mediated pneumonitis | Pneumonitis | Pulmonary | 2 | 3.6 | 12.5 |
| Immune-mediated myocarditis | Myocarditis | Cardiac | 1 | 1.8 | 6.3 |
| Endocrine toxicity | Adrenal insufficiency | Endocrine | 1 | 1.8 | 6.3 |
| **Total** | **—** | **—** | **16** | **28.6** | **100.0** |

Note: Percentages in the fifth column use all Score 2 patients as the denominator (n=56), whereas percentages in the sixth column use all severe irAEs as the denominator (n=16). Severe irAEs were graded according to CTCAE version 5.0. Abbreviations: irAE, immune-related adverse event; SII, systemic immune-inflammation index; PNI, prognostic nutritional index.
